# Draft Genome Sequence for a Urinary Isolate of *Nosocomiicoccus ampullae*

**DOI:** 10.1128/genomeA.01248-16

**Published:** 2016-11-17

**Authors:** Evann E. Hilt, Travis K. Price, Katherine Diebel, Catherine Putonti, Alan J. Wolfe

**Affiliations:** a Department of Microbiology and Immunology, Stritch School of Medicine, Health Sciences Division, Loyola University Chicago, Maywood, Illinois, USA; b Bioinformatics Program, Loyola University Chicago, Chicago, Illinois, USA; c Department of Biology, Loyola University Chicago, Chicago, Illinois, USA; d Department of Computer Science, Loyola University Chicago, Chicago, Illinois, USA

## Abstract

A draft genome sequence for a urinary isolate of *Nosocomiicoccus ampullae* (UMB0853) was investigated. The size of the genome was 1,578,043 bp, with an observed G+C content of 36.1%. Annotation revealed 10 rRNA sequences, 40 tRNA genes, and 1,532 protein-coding sequences. Genome coverage was 727× and consisted of 32 contigs, with an *N*_50_ of 109,831 bp.

## GENOME ANNOUNCEMENT

As part of an attempt to characterize the newly discovered female urinary microbiota (1–9), we report here the genome sequence and annotation of a strain of *Nosocomiicoccus ampullae* isolated from a female pursuing urogynecologic clinical care. This is the first report of a human isolate of *N. ampullae*, a species that has not been associated with pathogenesis.

*N. ampullae* strain UMB0853 was isolated from urine obtained by transurethral catheterization of an adult woman with urinary symptoms, using the described enhanced quantitative urine culture protocol (4). Strain UMB0853 was subcultured to purity and analyzed by matrix-assisted laser desorption ionization−time of flight mass spectrometry (10), which could not provide an assignment. In contrast, 16S rRNA gene sequence analysis identified the isolate as *N. ampullae*. A pure culture was stored at -80°C in a 2-ml CryoSaver brucella broth with 10% glycerol, and no beads (Hardy Diagnostics). For genome extraction and sequencing, the preserved pure culture isolate was grown on 5% sheep blood agar (BD BBL prepared plated medium) under 5% CO_2_ at 35°C for 48 h.

To extract genomic DNA, cells were resuspended in 0.5 ml of DNA extraction buffer (20 mM Tris-HCl, 2 mM EDTA, 1.2% Triton X-100 [pH 8]), followed by the addition of 50 µl of lysozyme (20 mg/ml), 30 µl of mutanolysin, and 5 µl of RNase (10 mg/ml). After a 1-h incubation at 37°C, 80 µl of 10% SDS and 20 µl proteinase K were added, followed by a 2-h incubation at 55°C. Two hundred ten microliters of 6 M NaCl and 700 µl of phenol-chloroform were added. After a 30-min incubation with rotation, the solutions were centrifuged at 13,500 rpm for 10 min, and the aqueous phase was extracted. An equivalent volume of isopropanol was added, and the solution was centrifuged at 13,500 rpm for 10 min after a 10-min incubation. The supernatant was decanted and the DNA pellet precipitated using 600 µl of 70% ethanol. Following ethanol evaporation, the DNA pellet was resuspended in Tris-EDTA (TE) and stored at -20°C.

Genomic DNA was diluted in water to a concentration of 0.2 ng/µl, as measured by a fluorometric-based method (Life Technologies); 5 µl was used to obtain a total of 1 ng of input DNA. Library preparation of the isolated DNA was performed using the Nextera XT DNA library preparation kit. Two libraries were prepared and sequenced during separate runs on the MiSeq sequencer (Illumina) using the MiSeq reagent kit version 2 (300 cycles). The two runs produced 11,221,884 reads in total. Assembly was performed using Velvet (11) (*k* = 99), followed by SSPACE (12) for scaffolding, producing 32 contigs, which varied from 2,642 bp to 337,531 bp (*N*_50_, 109,831 bp), with an average coverage of 727×. The NCBI Prokaryotic Genome Annotation Pipeline (13) detected 10 rRNA genes, 40 tRNA genes, 1,532 protein-coding sequences, and 35 pseudogenes. Six clustered regularly interspaced short palindromic repeat sequences (CRISPRs) were found (14). The genome size was 1,578,043 bp, with an observed G+C content of 36.1%.

### Accession number(s).

The draft whole-genome project for *N. ampullae* strain UMB0853 has been deposited at DDBJ/EMBL/GenBank under accession number MBFG00000000. Raw sequence reads are deposited at DDBJ/EMBL/GenBank under accession number SRR3828836.
